# Translocation, Rejection and Trapping of Polyampholytes

**DOI:** 10.3390/polym14040797

**Published:** 2022-02-18

**Authors:** Yeong-Beom Kim, Min-Kyung Chae, Jeong-Man Park, Albert Johner, Nam-Kyung Lee

**Affiliations:** 1 Department of Physics and Astronomy, Sejong University, Seoul 05006, Korea; kxc926@naver.com (Y.-B.K.); cmkk0820@naver.com (M.-K.C.); 2 Department of Physics, The Catholic University of Korea, Bucheon 14662, Korea; 3 Institut Charles Sadron (ICS), CNRS-Unistra, Université de Strasbourg, F-67000 Strasbourg, France; albert.johner@ics-cnrs.unistra.fr

**Keywords:** translocation, polyampholytes, drift-diffusion, probability distribution function, Monte Carlo simulation

## Abstract

Polyampholytes (PA) are a special class of polymers comprising both positive and negative monomers along their sequence. Most proteins have positive and negative residues and are PAs. Proteins have a well-defined sequence while synthetic PAs have a random charge sequence. We investigated the translocation behavior of random polyampholyte chains through a pore under the action of an electric field by means of Monte Carlo simulations. The simulations incorporated a realistic translocation potential profile along an extended asymmetric pore and translocation was studied for both directions of engagement. The study was conducted from the perspective of statistics for disordered systems. The translocation behavior (translocation vs. rejection) was recorded for all $2^{20}$ sequences comprised of *N* = 20 charged monomers. The results were compared with those for $10^{7}$ random sequences of *N* = 40 to better demonstrate asymptotic laws. At early times, rejection was mainly controlled by the charge sequence of the head part, but late translocation/rejection was governed by the escape from a trapped state over an antagonistic barrier built up along the sequence. The probability distribution of translocation times from all successful attempts revealed a power-law tail. At finite times, there was a population of trapped sequences that relaxed very slowly (logarithmically) with time. If a subensemble of sequences with prescribed net charge was considered the power-law decay was steeper for a more favorable net charge. Our findings were rationalized by theoretical arguments developed for long chains. We also provided operational criteria for the translocation behavior of a sequence, explaining the selection by the translocation process. From the perspective of protein translocation, our findings can help rationalize the behavior of intrinsically disordered proteins (IDPs), which can be modeled as polyampholytes. Most IDP sequences have a strong net charge favoring translocation. Even for sequences with those large net charges, the translocation times remained very dispersed and the translocation was highly sequence-selective.

## 1. Introduction

Polyampholytes (PAs) are polymers carrying positive and negative charges along their sequence. Most proteins are PAs. There is a special class of proteins which does not have a well-defined ground state. These intrinsically disordered proteins (IDPs) [1] are well-described by PA models [2,3]. The PAs-IDPs analogy also holds for disordered sequences in proteins with intrinsically disordered regions (IDRs) [4]. In high salt conditions, as implicitly considered below, or for large net charges, polyampholytes adopt open configurations provided their backbone is not too hydrophobic. The presence of charges of both signs along the sequence confers a specific translocation behavior to PAs and IDPs compared to the more widely studied polyelectrolytes [5,6,7,8,9,10,11], e.g., DNA, carrying negative charges only.

In nature, charged polymers (proteins, RNA) are imported in or exported from the cell nucleus by translocation through a nuclear pore [12]. Nuclear translocation is promoted by helpers [13] (importins, exportins) acting on (binding to) specific subsequences (labels) and is highly selective. Translocation through a single pore has been implemented in the laboratory [8], where translocation is typically driven by an electric field, mainly to study DNA sequence.

**Figure 1:**
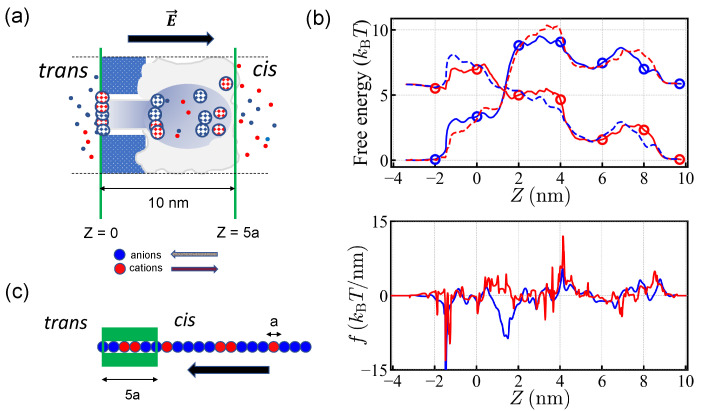
(**a**) Schematic representation of an α-hemolysin pore. The mushroom-shaped complex is approximately 10 nm long. The colored patches represent charged regions inside the pore, a positively charged *cis* protrusion and a negatively charged *trans* edge. (**b**) Translocation free energy (top) and corresponding translocation force (bottom) for small ions, K${}^{+}$ (red) and Cl${}^{-}$ (blue), in α-hemolysin, with a transmembrane potential of +150 mV (solid lines) and −150 mV (dashed lines). The potential is in favor of translocation of negative (positive) ions from *cis*-to-*trans* (reverse) direction with +150 mV. The free energy values are taken from Figure 7 of Ref. [22] and those values used for the MC simulations are indicated by circles. (**c**) Schematics showing the initial position of the PA chain in the MC simulation for translocation from the *cis* to the *trans* side.

The pores found in nature are classified into two types depending on their structure: symmetric ones such as porins, and asymmetric ones such as α-hemolysin and porin F (OmpF). α-hemolysin is related to diseases such as staphylococcus infection and is widely used for in vitro experiments. Typically such experiments apply electric potentials much larger than the cell transmembrane potential of ∼50–70 mV. α-hemolysin is a heptamer associated around the pore axis and its axial structure is rather complex (see Figure 1a). It has a (positively) charged *cis* protrusion, which favors the presence of (negatively) charged biopolymer sequences in the vicinity of the pore entry. The *trans* edge is negatively charged. Two regions, the *cis* vestibule and a *trans* channel stem, are separated by a narrow constriction, about 3 nm long and 1 nm wide, which is essentially polar. Translocation dynamics of negatively charged particles [14] and polymers (polyelectrolytes) through the α-hemolysin has been addressed [15,16,17]. Simulation studies include the influence of pore charge [18], heterogeneity in charge distribution [19], polymer rigidity [20], solvent conditions [21] and temperature [8].

The structure of the α-hemolysin pore is known at the scale of single residues. Realistic numerical simulations have been undertaken to elucidate the pore conductivity for small ions (Na${}^{+}$, K${}^{+}$, Cl${}^{-}$) especially for α-hemolysin, which is an asymmetric pore. The equilibrium concentrations of the salt ions along the pore axis were recorded and the associated free energy profile was obtained in [22,23]. The arrangement of the fixed charges located in pore walls entails a complex free energy profile (Figure 1b). Under the electric potential of +150 mV, applied to the *trans* side with respect to the *cis* side, positive charges tend to move from the *trans* side to the *cis* side while negative charges tend to move from the *cis* side to the *trans* side. (See, Figure 1a.) Due to the polarity and geometry of α-hemolysin, the free energy profiles are not symmetric under the inversion of translocation direction. Note that there is a free energy barrier for both types of charges moving from the *cis* side to the *trans* side. The translocation force is localized at the edge of the *trans* side for negative charges moving in the *cis*-to-*trans* direction, while positive charges moving in the reverse direction experience more or less uniform force over the extended pore.

Considering that the pore is a nanometer wide at the constriction, the polymer undergoing translocation is basically threaded monomer by monomer. Usually proteins can only translocate upon denaturation (not in their native state). Similarly, bulky ramified polymers [24,25], soft nanoparticles [26], vesicles [27] or associated micelles are sterically hindered [28] and either have to adopt energetically costly conformation or dissociate.

In previous studies, we addressed the effect of the charge disorder (randomness in charge arrangement along the sequence) in long sequences [29], by means of analytical theory, and the influence of the globular structure of PAs on their translocation through a point-like pore (of monomeric length) [30]. It was shown that a disorder in PA slows down the translocation dynamics driven by the overall favorable net charge.

In this work, the pore is modeled by a one-dimensional asymmetric free energy profile for anions and cations extending over several monomer sizes in length (Figure 1b). For an extended pore such as that considered here, the translocation force is correlated inside the pore as long as the content of the pore is not renewed. Furthermore, for short sequences, even physical quantities that self-average asymptotically, such as the translocation velocity, remain noisy.

We adopt the simple picture that the charges inside the pore experience the driving force endowed by the free energy of small ions and the polymers are basically one-dimensional sequence of charges (Figure 1c). This amounts to ignoring the variation of the extra entropy penalty of the polymeric sequence engaged for translocation and is reasonable as long as the pore is filled. It is also expected that the small ion distribution is disturbed by the electrical current. Similarly, the free energy of the polymeric charges may be influenced by translocation. Here we stick to the established static free energy profile.We also neglect the interactions between polymeric charges and the variation of the polymer density along the pore, which may matter in the *cis* vestibule where the polymer could coil. We consider open PAs, a situation expected at high ionic strength (molar) for backbones in theta or a good solvent. Such high ionic strength is often used in translocation experiments to ensure proper small ion conductivity.

The translocation of polyelectrolytes [6,7] in vitro is ensured by an external electric field. A theoretical study reports that the trapping of short polyelectrolytes may happen by dielectric trap and delay the translocation dynamics [31]. In contrast, the success of the translocation of the PA chains is strongly influenced by the charge sequence comprising antagonistic blocks and the translocation time is mainly determined by the disorder in the charge sequence.

We perform Monte Carlo (MC) simulations for the ensemble of sequences obtained by exact enumeration for *N* = 20 ($2^{20}∼10^{6}$ sequences) and for an ensemble of $10^{7}$ random sequences for *N* = 40. Exact enumeration addresses all sequences. This is important because a few specific sequences can strongly influence the statistics (moments of the translocation/rejection time distributions). We analyze the role of the net charge and disorder along the charge sequences under the realistic translocation potential profile of the α-hemolysin pore, when PAs are engaged into the asymmetric pore in either direction.

Most of the current work adopts the standpoint of statistics for disordered systems. We show the emergence of universal asymptotic features for the translocation statistics including the translocation time distribution. We provide a criteria for the successful translocation of a charge sequence. From the standpoint of proteins it is shown that IDPs sequences typically belong to the class of more easily translocating sequences.

In the following sections, we present the theory, the simulation model and results. Our MC results are rationalized by analytical arguments.

## 2. Theory

The translocation dynamics and the statistics of long sequences through a point-like pore have been addressed previously. These predictions for long sequences with a point-like pore will prove useful to rationalize our numerical findings. We present a short summary of earlier works [29,32] together with predictions for the escape time distributions of trapped sequences.

We assume that the charge statistics is uncorrelated along the chain. Each site carries a charge *q*, either +1 or −1, independently, with an average 〈q〉 and a variance $\sigma_{1}^{2}=1-{\langle q\rangle }^{2}$ (Note that $q^{2}=1$). Furthermore, the pore accommodates the charge one by one and is solely characterized by the translocation potential *U*, expressed in units of $k_{B}T$. The translocation motion of the chain results from a competition among thermal noise, the drift controlled by the force acting on the average charge per site 〈q〉 and potential barriers faced by antagonistic blocks. The height of the *typical* barrier $E_{b}$ built along a sequence due to *n* random charges increases with the charge fluctuation $n\sigma_{1}^{2}$ as $E_{b}=\sqrt{n}\sigma_{1}U$. The typical barrier due to the disorder in charge sequence is hence relevant for sequences longer than $n_{d}=1/\left(\sigma_{1}^{2}U^{2}\right)$, defined via $E_{b}∼1$. Because the drift makes the energy decrease linearly with *n*, the drift formally allows us to overcome the typical barrier when long enough part of the sequences, $n>n_{c}$, are already translocated. From the total energy barrier $E_{B}∼\sqrt{n}\sigma_{1}\left|U\right|-n\langle q\rangle U$, the crossover length $n_{c}$ is defined as $n_{c}+1=1/{\langle q\rangle }^{2}$ (by setting $E_{B}=0$). The typical barrier $E_{b}$ never becomes relevant if $n_{d}>n_{c}$, because the drift dominates over the disorder already in the thermal regime. The only parameter of the translocation process can be defined as $\mu =\sqrt{n_{d}/n_{c}}$. At the adopted qualitative level, the disorder along a long sequence matters for μ≲1. For an overall favorable translocation condition, U〈q〉<0, considered below, the scaling prediction for μ, $\mu ∼-\frac{\langle q\rangle }{U(1-{\langle q\rangle }^{2})}$, suggests that the disorder along a long charge sequence matters more at larger translocation potentials *U*. A quantitative theory [29,32] leads to the more precise definition of μ: 〈exp(−qμU)〉=1. The expression of μ, obtained from Gaussian statistics for the charge per site, coincides with the above scaling estimate for μ with the prefactor set to 2;

(1) $$\mu =-\frac{2\langle q\rangle }{U(1-{\langle q\rangle }^{2})}.$$

Let *n* be the length of the translocated part of the sequence at time *t*. In the absence of drift (〈q〉=0), the translocation follows the ultra slow Sinai dynamics [33]$\langle n^{2}\rangle ∼{\left(\log t\right)}^{4}$, see details in [34,35,36]. A similar slow regime is expected for $n_{d}<n<n_{c}$ in the disorder-dominated creep regime (μ<1). In the creep regime, the velocity does not behave well and $\langle n\rangle ∼t^{\mu }$. In the driven regime, at a lower disorder (1<μ<2), the velocity behaves well and 〈n〉∼t. However, the fluctuation, $\langle n^{2}\rangle -{\langle n\rangle }^{2}∼t^{2/\mu }$, stays anomalous. At a very low disorder (μ>2), both the velocity and diffusion constant are well defined but depend on μ. Ultimately, each engaged sequence is either translocated or rejected, albeit the waiting time may be arbitrarily long. The ratio of rejection probability to translocation probability is given by the splitting probability discussed in Appendix A. Disorder favors rejection even with favorable charge conditions. For Gaussian statistics, the average of the ratio of rejection probability to translocation probability over the disorder formally diverges for μ<1 and rejection strongly prevails.

If a sequence remains engaged in the channel for a long time, we consider it to be trapped [37] and it has to escape through energy barriers opposing both translocation and rejection. The distribution of sojourn times under a random uncorrelated force (the time distributions discussed here are not self-averaging) is discussed in [32]. At long times it behaves as $P_{\mathrm{sojourn}}\left(t\right)∼t^{-\mu }$. The distribution of relaxation times $P_{\mathrm{relax}}\left(t\right)$ under a random uncorrelated force decays as a power law asymptotically,

(2) $$P_{\mathrm{relax}}∼t^{-1-\mu }.$$

Qualitatively, this power law is the derivative of the one for the related sojourn probability. It can be loosely understood as the waiting time distribution in front of the trapping barrier. The translocation time distributions $P_{\mathrm{tr}}\left(t\right)$ and the rejection time distributions $P_{\mathrm{rej}}\left(t\right)$ are escape time distributions from traps built up along the sequences. In line with the regimes for the translocation dynamics, the power-law tail is integrable for μ>0, the first moment of the distribution is well defined for μ>1 and the second moment for μ>2. Note that the sojourn time distribution decays as an inverse power of logt in the Sinai regime (μ = 0). Long-time translocation/rejection may involve multiple trapping. The power law $∼t^{-1-\mu }$ is nonetheless expected for the translocation time distribution $P_{\mathrm{tr}}\left(t\right)$ and the rejection time distribution $P_{\mathrm{rej}}\left(t\right)$ when the dominant events are escapes from single trapping or when the translocation/rejection time is essentially prescribed by the deepest trap.

The power-law tail of the relaxation time distribution is integrable but, as mentioned above, formally, higher moments of the relaxation time distribution are not defined at a high disorder. Indeed the power law for an infinite sequence stems from the distribution of barriers of all heights. Strictly speaking, for a chain of finite length, the higher moments are well-behaved because of the cut off of the power law, as an effect of the finite sequence. Unfortunately, the translocation time involving the typical barrier is given by $\log t∼U\sqrt{N}$ for 〈q〉=0 and is difficult to reach in simulations even for finite sequences, resulting in trapped states. If we distinguish three final classes, translocated, rejected and trapped, the last class should be almost empty for a precise determination of the translocation statistics. For a finite sequence, the barrier height distribution is cut and the very tail of the time distribution is strongly depleted. In turn, the “experimental” waiting time must be long enough to reach deep in the cut-off regime to obtain the average translocation time of disordered sequences.

The large energy barriers are developed from blocky sequences. For 〈q〉=0, the symmetric diblock occurs with probability $1/2^{N}$ and, if properly engaged, faces the barrier UN/2 in both directions of translocation and rejection. This sole sequence hence contributes ∼exp(UN/2−Nlog2) to the average translocation time, which, provided U>2log2, can be very large. It is as large as the ∼$10^{20}$ monomeric diffusion times for *U* = 6 and *N* = 20. The situation is less dramatic for sequences with a favorable net charge. For a favorable net charge *Q*, the contribution of diblock sequences to the average translocation time decays exponentially with *N* provided 1−Q/N<2log2/U. For *U* = 6 this criterion gives Q/N>0.77. We distribute the sequences in classes with a prescribed net charge to study this issue.

## 3. Model: Monte Carlo Simulation

In the Monte Carlo (MC) simulations, we modeled the PA as a one-dimensional chain and explored the influence of the net charge and of the charge blocks in the PA sequences on their translocation.

The length of the α-hemolysin pore spans about 10 nm. Assuming that the effective monomer size carrying a unit charge (for protein it may cover 3-4 amino-acids) is about a∼ 2 nm, the translocation channel was modeled to be 5*a* long and thus 5 monomers can be put in the pore at the same time. During the translocation process, the 5 monomers were positioned 0, 2, 4, 6, and 8 nm away from the edge of the pore (Figure 1).

We considered MC hopping moves (of the whole chain) by the step size of monomeric length *a*. For each MC step, the possible move was randomly chosen to be +a or −a. The prescribed move was accepted following the standard Metropolis algorithm. As shown in Figure 1c, the 5 monomers were deployed in the pore at the start of each run of the simulations, the head monomer being at the edge of the *trans* side when engaging from the *cis* side to the *trans* side. For the direction from the *trans* side to the *cis* side, which is referred as the reverse direction below, we deployed the head monomer at the edge of the *cis* side. This initial condition circumvented the discussion of the entry mechanism given in [38,39,40]. The successful translocation meant that the tail of the chain left the pore completely. If the entire chain was retracted out of the pore, it was considered as rejection.

We took free energy values given for two types of charges at each site as shown in Figure 1b (see, also Table 1). For each step, the free energy difference of the whole chain was evaluated as the translocation process proceeded. The potential drop of *U* = 150 mV was applied over the pore [22] so that the free energy loss (gain) was ∼6 $k_{B}T$ per translocated favorable (antagonistic) charge. The applied polarity favored the translocation of anions. As noted earlier, the free energy profiles for anions and cations almost exactly exchange upon a change of polarity. We expected the translocation to be almost unchanged under the simultaneous changes of the polarity U→−U and sequence ±→∓. The free energy difference of the (MC) moves determined the translocation motion of a sequence and ultimately its probability of translocation/rejection.

The entropy of the polymer was expected to be somewhat different from that of small ions due to its connectivity. Nonetheless, as long as the pore remained filled, the corresponding entropy change was not relevant. We also assumed that the free energy changes outside of pore can be neglected.

We first considered PA chains composed of *N* = 20 monomers carrying either types of charges +1 or −1. We enumerated all possible $2^{20}$ sequences, and for each sequence 100 independent trials for translocation were conducted. To explore the influence of the sequence length, we also tested the translocation times for *N* = 40, for which we created $10^{7}$ independent random sequences. The number of sequences was large enough to sample the bulk of the translocation/rejection time distributions investigated in this contribution. We introduce the finite waiting time, that is, $1.6\times 10^{5}$ or $10^{6}$ MCT. The translocation dynamics can be ultra-slow for some blocky sequences. The sequences qualified for successful translocation should satisfy a certain rule in charge arrangement.

## 4. Results and Discussion

**Figure 2:**
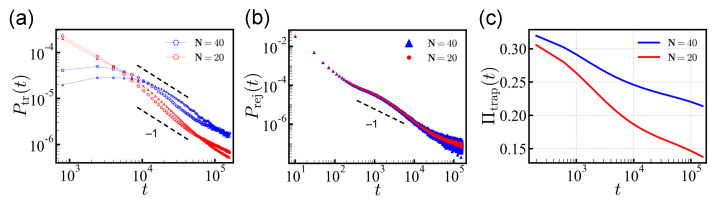
(**a**) The translocation time distributions for 2${}^{20}$ sequences of *N* = 20. The number of translocated sequences are measured in the time interval [t−δt/2, t+δt/2] with δt = $1.6\times 10^{3}$ MCT. The symbols ○ and Δ represent the *cis*-to-*trans* and reverse directions, respectively. (**b**) Distributions of rejection times, measured within the time interval [t−δt/2, t+δt/2] with δt = 20 MCT. Dashed lines are guides for the power-law relation, $P\left(t\right)∼t^{-1}$. (**c**) Logarithmic decay of trapped populations, $\Pi_{\mathrm{trap}}\left(t\right)$, normalized by the total number of translocation trials.

We obtained the probability distribution functions (PDFs) of translocation times $P_{\mathrm{tr}}\left(t\right)$ and rejection times $P_{\mathrm{rej}}\left(t\right)$ from the global ensemble of all $2^{20}$ sequences, which satisfies 〈Q〉 = 0 by symmetry. We measured the number of translocated sequences within the given binning time, δt = $1.6\times 10^{3}$ MC time steps (MCT), for both *cis*-to-*trans* and the reverse directions, and the distribution was normalized by the total number of successful attempts. As predicted from theory, we found that the power-law tail $P\left(t\right)∼1/t^{1+\mu }\left(\mu =0\right)$ indeed prevailed asymptotically. (Figure 2a) For *N* = 20, the PDF $P_{\mathrm{tr}}\left(t\right)$ deviated from the power law for $t≳10^{4}$ MCT (more pronounced for the *cis*-to-*trans* direction) and dropped more quickly because of the finite-length effect. As we show later, the deviation from the $t^{-1}$ behavior was due to the relatively fast translocating sequences carrying large net charges *Q*. These effects appeared over a limited intermediate time interval and the PDF recovered $t^{-1}$ behavior for long times, $t>4.0\times 10^{4}$ MCT.

We also show the translocation times of $10^{7}$ independent random sequences of *N* = 40 in Figure 2a. The PDF $P_{\mathrm{tr}}\left(t\right)$ of *N* = 40 also follows $t^{-1}$ for times $t>10^{4}$ MCT. At short times, the sequences with *N* = 40 show a markedly flat translocation distribution in contrast to the *N* = 20 case. The highly charged sequences with the shorter length *N* = 20 translocate typically without significant barrier. However, for the longer sequences, *N* = 40, significant barriers can be developed more frequently. In addition, sequences of the same charge density Q/N carry a doubled net charge and are statistically less common in the ensemble of *N* = 40 as compared to *N* = 20. This is because the standard deviation of the total net charge (*Q*) distribution only increases sub-linearly, as $\propto \sqrt{N}$.

The rejection probability is also shown in Figure 2b. The rejection time distribution $P_{\mathrm{rej}}\left(t\right)$ has two regimes: The short-time behavior, which is determined by the sequence of five head monomers, and the long-time behavior that is governed by the distribution of energy barriers against rejection. Once the chain is trapped, the move in either *cis/trans* direction requires to overcome similar energy barriers, and results in a power-law tail similar to $P_{\mathrm{tr}}\left(t\right)$.

For the translocation of *N* = 20, with the waiting time $t_{w}$ = $1.6\times 10^{5}$ MCT, the population of successfully translocated/rejected sequences was 9.3% (9.1%)/76.9% (74.6%), in the *cis*-to-*trans* (reverse) direction, respectively. (note that there were 16% of sequences with a neutral net charge and 43% each with a favorable/antagonistic net charge) Some sequences, 13.8% (16.3%), were found to be trapped in the pore. As shown in Figure 2c, the population of the trapped sequences $\Pi_{\mathrm{trap}}\left(t\right)$ only decreases logarithmically over time, and some sequences remain undetermined even after a long simulation time. Here we classified the trapped sequences separately.

For translocations in the *cis*-to-*trans* (reverse) direction of longer *N* = 40 sequences, the proportion of successful translocation became considerably smaller ∼1.6% (1.0%) but the rejection rate was almost the same with $t_{w}$ = $1.6\times 10^{5}$ MCT. A significant fraction of sequences ∼21.4% (24.4%) remained undetermined.

Despite the qualitative similarity in translocation behavior, when moving from the *cis*-to-*trans* side, there were less cases of trapping and slightly more cases of both translocation and rejection. For the longer waiting time $t_{w}$ = $10^{6}$ MCT, the successful translocations were 11.4% (10.9%) of the total population of *N* = 20 for the *cis*-to-*trans* (reverse) direction and the trapped sequences decreased to 9.7% (12.1%). The results are summarized in Table S1 in Supplementary Materials.

**Figure 3:**
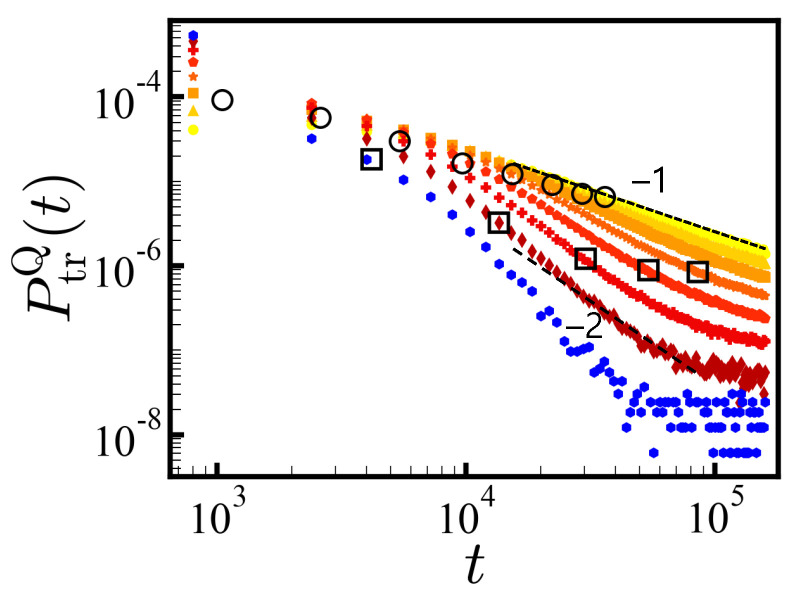
Distributions of translocation times (in the reverse direction) for various *Q*-ensembles of *N* = 20. The PDF measures the fraction of translocated sequences at given time interval [t−δt/2, t+δt/2] with δt = 1600 MCT for each *Q*-ensemble. Each distribution is normalized by the total number of successfully translocated sequences with $t_{w}$ = $1.6\times 10^{5}$ MCT. Colors from yellow to blue represent charge values of *Q* = 0, 2, 4, 6, 8, 10, 12 and 14, respectively. The dashed lines are a guide for the eyes indicating power-law relations, $P\left(t\right)∼t^{-(1+\mu )}$ with μ=0 and 1. For each *Q*, we indicate the average translocation times $\langle t_{\mathrm{tr}}\rangle$ by ○. The $\langle t_{\mathrm{tr}}\left(Q\right)\rangle$ decreases with increasing net charges. The square symbols for *Q* = 6, 8, 10, 12 and 14 represent the average translocation times $\langle t_{\mathrm{tr}}\left(Q\right)\rangle$ with $t_{w}$ = $10^{6}$ MCT.

**Figure 4:**
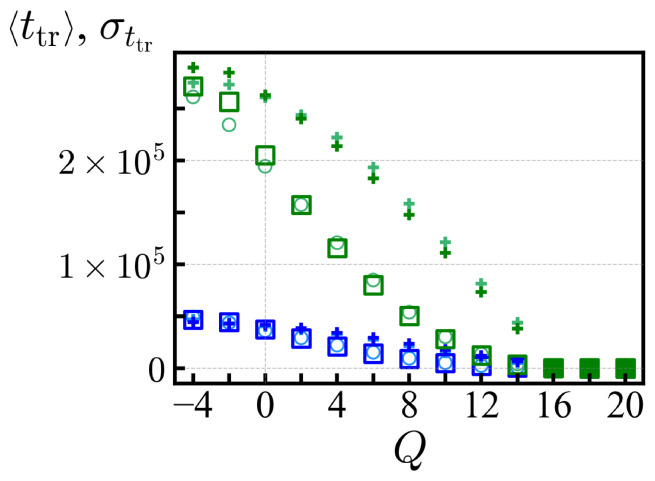
The average translocation times $\langle t_{\mathrm{tr}}\rangle$ in the *cis*-to-*trans* (□) and reverse (○) directions as a function of the net charge *Q* for *N* = 20. The + symbols represent the standard deviations of the corresponding data. The averages are obtained from the successful translocation trials with waiting times $t_{w}=1.6\times 10^{5}$ (blue) and $10^{6}$ (green) MCTs, respectively. The filled symbols (Q>15) indicate the convergence of data independent of the waiting time.

The translocation behavior was mostly determined by the charge arrangement in sequences, and for all times, the statistical errors in the translocation/rejection probability was negligibly small, ∼0.03%–0.07%, as reflected in the digit. However, the disorder in the charge sequences caused large variance among sequences in translocation times. We discuss this point further below. (See Figure 3 and Figure 4.)

Because some quantities, such as average translocation times, are not well defined due to the unresolved sequences, we compared the time required for a population of the same size to translocate in each direction (Table 2). Clearly, translocation is more efficient in the *cis*-to-*trans* direction. This is because a large translocation force is localized when engaging in the *cis*-to-*trans* direction. See also Figure 1b for the translocation force.

A large favorable net charge *Q* promotes translocation. We classified the sequences according to their net charges. The translocation behavior was qualitatively similar in both directions, so we present results obtained in one direction only unless explicitly mentioned. Results for both directions of translocation are shown in Supporting Information (SI) as a supplement. Hereafter and in SI, we represent +1 and −1 for favorable and antagonistic types of charges from the view of translocation.

In Figure 3, we show the PDFs of translocation times (in the reverse direction) in log–log scale for each *Q* separately (*N* = 20) with $t_{w}$ = $1.6\times 10^{5}$ MCT (for *cis*-to-*trans*, see SI). For the ensembles of sequences with net charge *Q*, the average translocation force increases with *Q* resulting in a steeper decay of the PDF of translocation times. Each PDF $P_{tr}^{Q}\left(t\right)$ follows the power law $∼t^{-(1+\mu )}$ with μ<1 at times before the finite size effect sets in. The larger the net charge, the steeper the slope −(1+μ) is, as discussed in the theory section. A fit of the exponent μ obtained from the Gaussian statistics needs to set 〈q〉 to Q/N and apply uncorrelated charge statistics. This is reasonable at low *Q* and indeed works fairly up to *Q* = 8 for *N* = 20 (see Figure 3 and Equation (1)), and up to *Q* = 16 for *N* = 40 (not shown). The subensemble of *Q* = 12 shows a $∼t^{-2}$ decay. Formally, the μ = 1 criterion for infinite chains and a point-like pore separate the disorder-dominated regime from the driven regime where the first moment of the PDF is well defined.

Since most of the large *Q* sequences are either translocated or rejected in a short time, for long times, the global dynamics shown in Figure 2 is dominated by the flux of small *Q* sequences that are slowly translocating.

For each ensemble of net charge *Q*, we obtained $\langle t_{\mathrm{tr}}\left(Q\right)\rangle$ with $t_{w}$ = $1.6\times 10^{5}$ MCT from the successfully translocated sequences. The average value of $\langle t_{\mathrm{tr}}\left(Q\right)\rangle$ decreased with increasing *Q* as indicated by the circles in Figure 3. Aforementioned, this average translocation time must be taken with care as long as trapped sequences are present. The released sequences after the given waiting times may greatly affect the average translocation time.

In Figure 4, we show the average translocation time $\langle t_{\mathrm{tr}}\left(Q\right)\rangle$ and its standard deviation $\sigma_{t_{\mathrm{tr}}}$ for each ensemble of net charge *Q* with *N* = 20, obtained with $t_{w}$ = $1.6\times 10^{5}$ and $10^{6}$ MCTs. Those *Q* values with vanishing trapped sequences and a steep decay of the PDF (say with a long-time slope steeper than −2, Q>15) provide a reliable estimate of the average translocation time and those cases are represented by filled symbols in Figure 4. For Q<15, the large standard deviation $\sigma_{t_{\mathrm{tr}}}$ reflects the presence of large antagonistic blocks, and the statistics of translocation time as measured here is influenced by the waiting time. For Q>15, the mean translocation time $\langle t_{\mathrm{tr}}\rangle$ is independent of the waiting times $t_{w}$ and converges to a value much smaller than $t_{w}$. The criterion Q>15 corresponds to the value of net charges where the large free energy barrier of diblock sequence does not dominate the average $\langle t_{\mathrm{tr}}\rangle$ (see Section 2). Translocation is selective with respect to the specific arrangement of the sequence (see also Table 3 below). On this account, the standard deviation $\sigma_{t_{\mathrm{tr}}}$ for *Q* = 16 remains larger than the sequence average $\langle t_{\mathrm{tr}}\rangle$, e.g., $\langle t_{\mathrm{tr}}\rangle \approx$ 180 (300) MCT and $\sigma_{t_{\mathrm{tr}}}\approx$ 500 (960) MCT for the *cis*-to-*trans* (reverse) direction. For *Q* = 18, the disorder resides in the position of a single antagonistic charge and does not contribute much to $\sigma_{t_{\mathrm{tr}}}$. Further discussions are available in Appendix B.

Below we discuss the control parameters for translocation, rejection and trapping.

**Figure 5:**
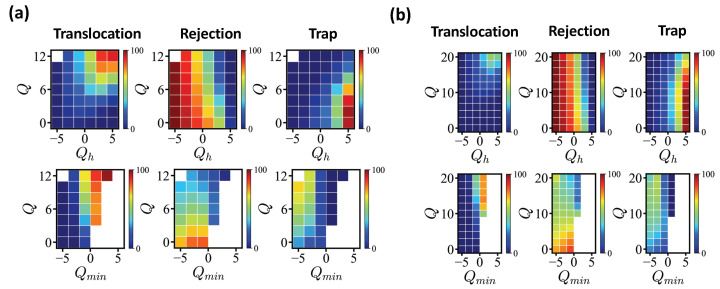
The percentages of translocated/rejected/trapped sequences (in the reverse direction) with $t_{w}$ = $1.6\times 10^{5}$ MCT (**a**) for exactly enumerated *N* = 20 sequences and (**b**) for $10^{7}$ randomly created sequences of *N* = 40. The top panels show the dependencies on $Q_{h}$ and *Q* and the bottom panels show the dependencies on $Q_{min}$ and *Q*. Color codes are presented in neighboring color bars.

We investigated the influence of the head sequence on translocation/rejection. Figure 5 (a) shows the fractions of translocated/rejected/trapped sequences for exactly enumerated *N* = 20 sequences with $t_{w}$ = $1.6\times 10^{5}$ MCT. As shown in the figure, the entry is first filtered by the net charge of the head sequence $Q_{h}$, counting the net charge of the first five monomers of the translocating edge. Sequences carrying $Q_{h}<0$ are mostly rejected and the larger the $Q_{h}$, the less likely a sequence is rejected. Once a sequence succeeds in entering, a sequence with a sufficiently large total net charge manage to translocate. Sequences with Q≳6 ($Q_{h}>0$) show translocation rate more than 50%. Less charged sequences are often trapped, at least for the given simulation time (data with $t_{w}$ = $10^{6}$ MCT are qualitatively similar). The translocation rate shows strong correlation with the number of antagonistic charges in the pore. We introduced as a control parameter the minimum net charge $Q_{min}$ measured for a block of pore length 5a along the sequence. If there are more antagonistic charges in the pore, the energy barrier increases accordingly. Sequences with $Q_{min}>0$ mostly pass successfully. Under this operational criterion, the formation of any high-energy barrier is not allowed. This is a sufficient but not a necessary condition (see for example Table 3).

We also conducted the same analysis for *N* = 40 sequences (Figure 5b), where we showed *Q* ranging from 0 to 20. The same waiting time $t_{w}$ = $1.6\times 10^{5}$ MCT applied. Similar to short sequences, the rejection was mainly controlled by the head sequence and the condition $Q_{min}>0$ held for a successful translocation. Only highly charged sequences (Q∼ 20) reached a translocation rate of more than 50%.

We compared the influence of $Q_{h}$ and $Q_{min}$ for *N* = 20 and *N* = 40, with the same 〈q〉 = 1/2. The rejection rates were 55.16%, 16.64% and 0.26% for *N* = 40 with $Q_{h}$ = 1, 3 and 5 of favorable head sequences. The impact of favorable $Q_{h}$ was somewhat reduced compared to *N* = 20, where the corresponding rejection rates were 44.61%, 14.84% and 0.38%. The successful translocation rates were considerably reduced to 32.71%, 50.00%, and 45.54% for *N* = 40 compared to 54.15%, 77.42% and 75.77% for *N* = 20 with the same $Q_{h}$ = 1, 3 and 5. These rates were reproduced with statistical errors ∼0.003–0.008%. The number of digits shows the accuracy of the data. As the chance to build a higher energy barrier was expected to grow significantly with the sequence length, the proportion of trapped/rejected states also increased significantly for *N* = 40, compared to *N* = 20.

Furthermore, we noted that the sequences with the largest value of $Q_{h}$ = 5 and with moderate net charges 〈q〉=Q/N<1/4 were mostly trapped (>95%) for both lengths due to the longer antagonistic block followed by. For sequences with 〈q〉=1/2 and $Q_{h}$ = 5, the fraction of trapped sequence was over 50% for *N* = 40 and ∼24% for *N* = 20.

For the given values of $Q_{min}$ = 1,−1 and −3, the translocation rates were 81.38%, 60.53%, and 7.30% for *N* = 20 and 75.00%, 46.04%, and 3.60% for *N* = 40, respectively. Summarizing, the net charge of head block $Q_{h}$ had a stronger impact on short sequences, but the success rate was mainly governed by the largest energy barrier, reflected by $Q_{min}$.

For the quantitative analysis, we compared the translocation behavior of several specific sequences with the prescribed net charge *Q* = 8 in Table 3. The average translocation time $\bar{t_{\mathrm{tr}}}$ and standard deviation $\sigma_{th}$ were obtained from 10,000 different trials for the given sequence, and $\bar{\cdots }$ and $\sigma_{th}$ denote the thermal averages and corresponding standard deviations. For each given sequence, the translocation statistics was well-defined and $\sigma_{th}<\bar{t_{\mathrm{tr}}}$, which contrasted with the noisy statistics of translocation times in the ensemble of disordered sequences. We may recall the translocation time statistics (*cis*-to-*trans*) for the subensemble of sequences with *Q* = 8, where we found the sequence average $\langle t_{\mathrm{tr}}\rangle ∼$ 8900 MCT and $\sigma_{\mathrm{tr}}=$ 23,500 MCT. The fastest translocating sequence was the regular sequence with a large $Q_{min}$ value. A block of two consecutive antagonistic charges (-1-1) corresponded to $Q_{min}$ = 1. When comparing sequences with traps with $Q_{min}$ = 1, the translocation time increased approximately in proportion to the number of traps. Note also that, despite the same condition of *Q* and $Q_{min}$, the translocation time also depended on the position of antagonistic blocks. For the sequences carrying a block of three antagonistic charges, (-1-1-1), they translocated successfully when $Q_{h}$ was favorable, whereas they tended to be trapped when $Q_{h}$ was less favorable.

**Figure 6:**
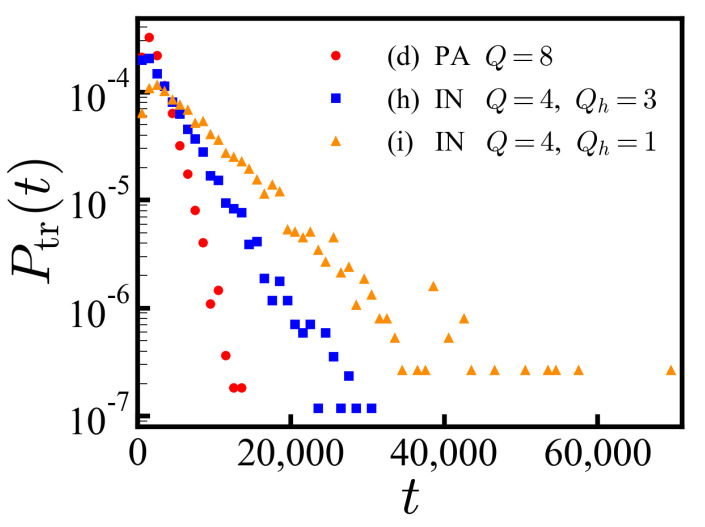
The PDFs of translocation times of PA (sequence (d) in Table 3) and IDP IN sequences (sequence (h) and (i) in Table 3) engaging in the *cis*-to-*trans* direction with $t_{w}$ = $1.6\times 10^{5}$ MCT. The number of translocated sequences are measured in the time interval [t−δt/2, t+δt/2] with δt = $1.0\times 10^{3}$ MCT. The PDFs of sequence (d) and (h) clearly show exponential decay as a function of time.

The last two sequences in Table 3 represent the sequence of protein IN from each end. We show only the charged residues ignoring neutral residues for simplicity, labeling the antagonistic charge type as −1. The PDF of translocation times for a specific IDP sequence (h) shows an exponential decay as a function of time (Figure 6), where we expect $\sigma_{th}\approx \bar{t_{\mathrm{tr}}}$. The statistic is well defined in contrast to the ensemble of disordered sequences. The exponential decay is less obvious for sequence (i) engaging from the opposite end.

## 5. Conclusions

We investigated the role of the disorder in the charge-sequence of polyampholyte chains on their translocation behavior. We considered an α-hemolysin pore under an electric field which is a widely used setup in in vitro experiments. By means of Monte Carlo simulations incorporating a realistic translocation potential profile along the pore, the translocation behavior (translocation vs. rejection) was recorded for all 2${}^{20}$ sequences comprised of 20 charged monomers by exact enumeration. The results were compared with those of an ensemble of $10^{7}$ random sequences comprised of 40 charges. In contrast to polyelectrolytes carrying one type of charges, the translocation dynamics of PAs are conserved by the disorder in the charge sequences. Even if PA has a net charge favorable for translocation, antagonistic barriers can be built up due to charge fluctuations along the sequence, leading to the PA trapping. The probability distributions of translocation/rejection times revealed a net charge dependence, power-law tails, and pore structure dependence. The short-time behavior in part depended on the initial conditions yet rejection was mainly controlled by the charge distribution of the head-sequence. Late-time translocation/rejection was governed by the escape from a trapped state over an antagonistic free energy barrier and the tails of both rejection and translocation time PDF were characterized by a power-law decay. Our findings were rationalized by the sojourn time probability in the potential created by a random uncorrelated force.

The slowly decaying power-law tail of the translocation time distribution makes the determination of the moments a difficult task. Numerically, the estimates of the moments will, in many cases, be influenced by the waiting time. For a subensemble of sequences with a large net charge, it is possible to estimate the average translocation time in the simulation, despite the noisy statistics in translocation times with an estimated standard deviation as large as the average.

With the translocation potential at hand, inverting the polarization is (almost) equivalent to reversing the sign of the charges along the sequence. However, inverting the direction of translocation and the polarization or the sign of the charges along the sequence is not equivalent, which reflects the asymmetric pore structure. Translocations in both directions remain, to a large extent, qualitatively similar, but the efficiency of translocation is different. Furthermore, the asymptotic laws are approached differently. This is also expected for other asymmetric pores.

In this contribution, we did not address concentration effects, including PA aggregation. PAs are less likely to aggregate at high salt concentrations (∼2 M) commonly used for translocation experiments. The formation of (binary) complexes before/after translocation can inhibit/promote translocation. This is reminiscent of importins/exportins in nuclear translocation.

We also devised simple criteria for the high translocation rate of a sequence. The head block filling the channel should be favorable or neutral to avoid immediate rejection. If every block filling the pore, which arises along the sequence, is also favorable or neutral, translocation is almost ensured as no high barrier can develop. This sheds some light on the selectivity of the translocation process.

In view of this work, we reconsider the two IDP sequences presented in [2,29]: The weekly charged IN and the highly charged PRoTα. Considering only charged residues, the IN protein counts 16 charges with a net charge of −4; its six positive (antagonistic) charges are dispersed along the sequence with the longest blocks of two consecutive positive charges. The PRoTα protein counts 63 charges with a net charge of −43. The 10 positive charges are dispersed along the sequence with longest blocks of three consecutive positive charges. Each IDP sequence exhibits well-defined translocation statistics. IDPs seem to disperse their minority charge along the sequence, which allows their plasticity. Typical IDP sequences do not comprise long (antagonistic) charge blocks and belong to the class with fast translocation/rejection, as introduced in our study.

## Figures and Tables

**Table 1:** Free energy values of ions relative to free solution. The positions in the pore are indicated in Figure 1.

Position	−a	0	*a*	2a	3a	4a	5a
Free energy of cations ($k_{B}T$)	5.53	6.98	5.00	4.65	1.59	2.32	0.07
Free energy of anions ($k_{B}T$)	0.07	3.35	8.82	9.08	7.47	7.00	5.87

**Table 2:** Comparison of translocation times for the given populations of *N* = 20 engaging in the *cis*-to-*trans* and reverse directions. The time resolution is given by the binning size δt = 400 MCT.

Translocated Population	1%	2%	4%	6%	8%	10%
$t_{\mathrm{tr}}$ (MCT), *cis*-to-*trans*	400	800	2400	7200	35,600	300,000
$t_{\mathrm{tr}}$ (MCT), reverse	400	800	3600	9600	39,200	436,000

**Table 3:** Comparison of translocation times of specific sequences with *Q* = 8 and *N* = 20 (a–g) and sequences of IDP IN (h,i) with *Q* = 4 and *N* = 16, engaging in the *cis*-to-*trans* direction under +150 mV of electric potential. (Favorable and antagonistic charges are labeled as 1 and −1, respectively.) Antagonistic charges are highlighted as red. The statistics were obtained with $t_{w}=1.6\times 10^{5}$ from 10,000 different translocation trials for each sequence. (a) Regular sequence, (b) 1 block of (-1-1), (c) reverse sequence of (b), (d) 2 blocks of (-1-1), (e) 3 blocks of (-1-1), (f) 1 block of (-1-1-1), (g) reverse sequence of (f), (h) sequence of IN, (i) sequence of IN, reverse of (h).

	Sequences	$Q_{\min}$	$Q_{h}$	$\bar{t_{\mathrm{tr}}}$ (MCT)	$\sigma_{\mathrm{th}}$ (MCT)	Success Rate (%)	Trapped Rate (%)
a	11-111-111-111-111-111-111	1	3	66	18	86	0
b	11-1-11111-1111-111-11-111	1	1	1050	860	55	0
c	11-11-111-1111-11111-1-111	1	1	340	170	66	0
d	11-1-11111-1-111-111-11111	−1	1	2250	1590	54	0
e	11-1-1111-1-11111-1-111111	1	1	4270	2490	31	0
f	111-1-1-111-11111-11111-11	−1	1	69,740	45,450	34	47
g	111-11111-1111-1111-1-1-11	−1	3	1440	1280	99	0
h	11-1111-1-11 -111-1-111	−1	3	3670	3460	84	0
i	11-1-111-11-1-1111-111	−1	1	7070	6360	37	0

## Data Availability

Not applicable.

## Supplementary Materials

The following supporting information can be downloaded at: https://www.mdpi.com/article/10.3390/polym14040797/s1, Table S1: The populations of translocation, rejection and trapping in both directions.

## Appendix A

Neutral sequences engaged in the pore are mostly rejected whereas those carrying a large favorable charge are mostly translocated. In the continuous limit, the ratio of rejection over translocation is given by $P_{\mathrm{split}}=\int_{0}^{\infty }a^{-1}dL\exp\left(U\sum_{i=0}^{L/a}q_{i}\right)$ which, when *q* is averaged over the Gaussian disorder, reduces to:

(A1) $$\langle P_{\mathrm{split}}\rangle =\int_{0}^{\infty }dLa^{-1}\exp\left\{(U\langle q\rangle +U^{2}\sigma_{1}^{2}/2)L/a\right\}.$$

Rejection is always promoted by the disorder. If U〈q〉>0, the drift is antagonistic and further favors rejection. With U〈q〉<0, the drift favors translocation and the actual behavior $\langle P_{\mathrm{split}}\rangle =\int_{0}^{\infty }dLa^{-1}\exp\left(\left(U\langle q\rangle \right)\left(1-\frac{1}{\mu }\right)L/a\right)$ is ruled by the parameter μ comparing the weight of the disorder and the drift. The integral diverges for μ<1 where the rejection probability is 1 (for the asymptotically long polyampholytes). For a weak disorder, μ>1, the averaged probability ratio is finite and given by $\langle P_{\mathrm{split}}\rangle =-\frac{1}{U\langle q\rangle (1-1/\mu )}$.

## Appendix B. The Dependency on the Waiting Time

As the nature of the sequence is reflected in the rate of successful translocation or trapping at any given time, the dependency on the waiting time was investigated for the given *Q*-ensemble. Sequences with small net charge *Q* were more likely trapped. For *Q* = 14, as we increased the waiting time from $1.6\times 10^{5}$ MCT to $10^{6}$ MCT, the trapped sequences were reduced from 0.9% to 0.3%, and translocated sequences increased from 89.3% to 89.8%. With *Q* = 0, the trapped sequences were reduced from 19.7% to 15.5%, and translocated sequences increased from 1.6% to 2.4%. Among the sequences released from the trapped state, more sequences were translocated (rather than rejected) for Q≥ 4, while for small net charges *Q* = 0 and 2, we found that, more sequences were rejected.
