# Supplementary material for: Translocation, Rejection and Trapping of Polyampholytes
Source: Polymers (Basel). 2022 Feb 18;14(4):797. doi: 10.3390/polym14040797 (PMC8877523; doi:10.3390/polym14040797)
Supplement: Supplementary file 1 [file polymers-14-00797-s001.zip › polymers-1586819-supplementary.pdf]

Supporting Information
Translocation, rejection and trapping of polyampholytes

Yeong-Beom Kim, Min-Kyung Chae,[1] Jeong-Man Park,[2, a)] Albert Johner,[3, b)] and Nam-Kyung Lee[1, c)]

[1)] *Department of Physics and Astronomy, Sejong University, Seoul 05006, Korea*

[2)] *Department of Physics, The Catholic University of Korea, Bucheon 14662, Korea*

[3)] *Institut Charles Sadron CNRS-Unistra, 23 Rue de Loess, 67034 Strasbourg CEDEX 2, France*

[a)] Electronic mail: jmanpark@catholic.ac.kr

[b)] Electronic mail: albert.johner@ics-cnrs.unistra.fr

[c)] Electronic mail: lee@sejong.ac.kr

A. Distributions of translocation times in *cis*-to-*trans* direction

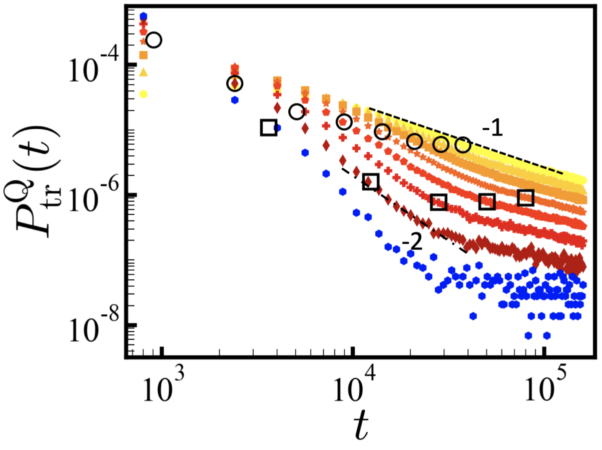

Figure S1. Distributions of translocation times in *cis*-to-*trans* direction for various Q-ensemble of $N = 20$. The PDF measures the fraction of translocated sequences at given time interval $[t - \delta t/2, t + \delta t/2]$ with $\delta t = 1600$ MCT for each Q-ensemble. Each distribution is normalized by the total number of successfully translocated sequences with $t_w = 1.6 \times 10^5$ MCT. Colors from yellow to blue represent values of favorable net charges $Q = 0$, 2, 4, 6, 8, 10, 12 and 14, respectively. The dashed lines are the guide for eyes indicating power law relation, $P(t) \sim t^{-(1+\mu)}$ with $\mu = 0$ and 1. For each Q, we indicate average translocation times $\langle t_{tr} \rangle$ by $\circ$. The $\langle t_{tr}(Q) \rangle$ decreases with increasing net charges. The square symbols for $Q = 6$, 8, 10, 12, 14 represent average translocation times$\langle t_{tr}(Q) \rangle$ with total MC time 10^6 MCT.

B. Translocation, rejection, and trapping in *cis*-to-*trans* and reverse directions

We compare translocation behavior in both directions for exactly enumerated $N = 20$ sequences and for random $N = 40$ sequences.

Table S1. The populations of translocation, rejection and trapping in both directions. Total number of sequences in the tested ensembles are 2^{20} for $N = 20$ and 10^7 for $N = 40$.

N (t_w)	direction	translocation(%)	rejection(%)	trapping(%)
$N = 20$	*cis*-to-*trans*	9.3	76.9	13.8
($t_w = 1.6 \times 10^5$)	reverse	9.1	74.6	16.3
$N = 40$	*cis*-to-*trans*	1.6	77.0	21.4
($t_w = 10^6$)	reverse	1.0	74.6	24.4
$N = 20$	*cis*-to-*trans*	11.4	78.9	9.7
($t_w = 1.6 \times 10^5$)	reverse	10.9	77.0	12.1

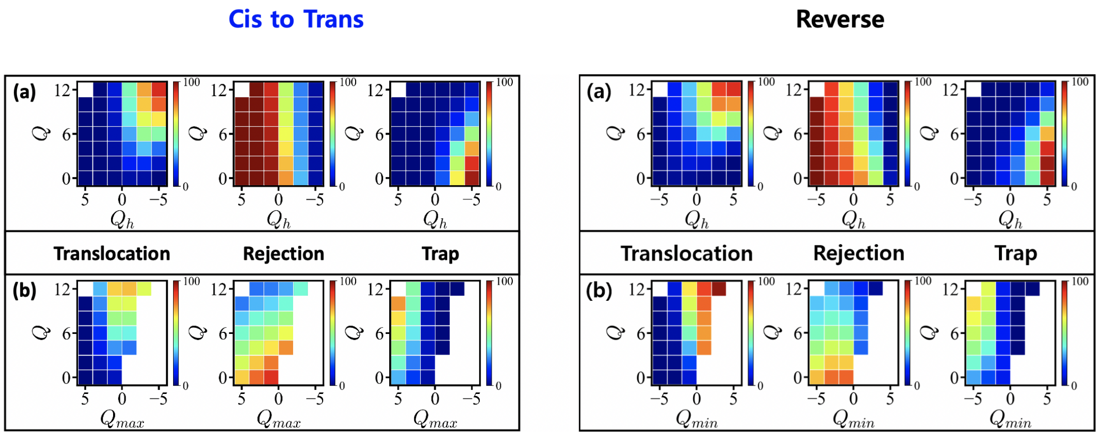

Figure S2. $N = 20$. The percentages of translocated/rejected/trapped populations are measured with $t_w = 1.6 \times 10^5$ MCT. The left panel shows data in *cis*-to-*trans* direction and the right panel shows the data in the reverse direction. Upper panels show the data for the given net charge of head sequence Q_h and Q and lower panels show the data for the minimum net charge Q_{min} of the segment of length $5a$ and Q. Color codes are displayed in neighboring color bars.

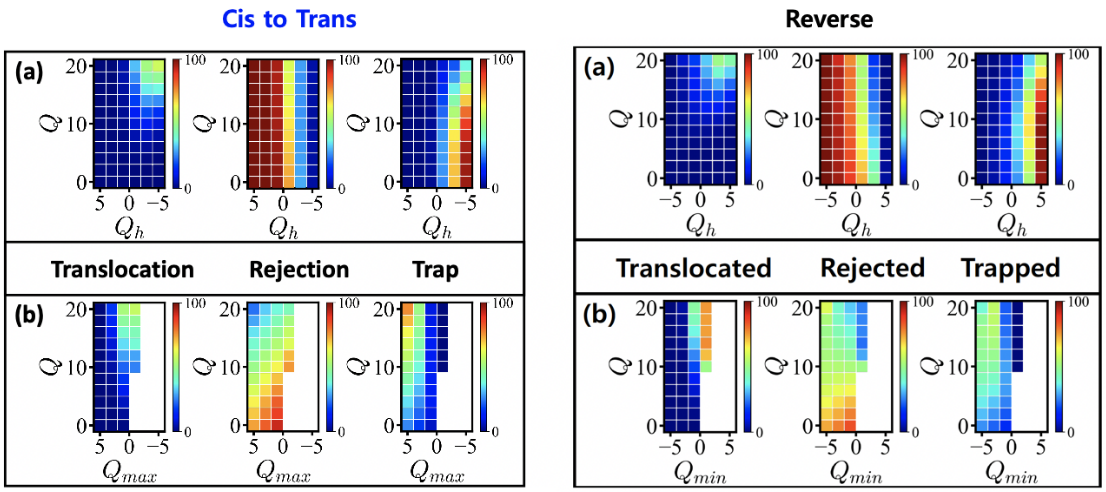

Figure S3. $N = 40$. The percentages of translocated/rejected/trapped populations are measured at $t_w = 1.6 \times 10^5$ MCT. The left panel shows data in *cis*-to-*trans* direction and the right panel shows the data in the reverse direction. Upper panels show the data for the given net charge of head sequence Q_h and Q and lower panels show the data for the minimum net charge Q_{min} of the segment of length $5a$ and Q. Color codes are displayed in neighboring color bars.
